# Supplementary material for: An open-source framework for large-scale, flexible evaluation of biomedical text mining systems
Source: J Biomed Discov Collab. 2008 Jan 29;3:1. doi: 10.1186/1747-5333-3-1 (PMC2276192; doi:10.1186/1747-5333-3-1)
Supplement: Additional file 1 — Evaluation platform source code. The additional file contains the software evaluation framework discussed in this paper. The most current release can be downloaded from SourceForge.net [1]. See the accompanying README file for instructions on installation and use. Also included in the distribution is a collection of UIMA wrappers for some commonly used BioNLP tools and annotated corpora. [file 1747-5333-3-1-S1.zip › bionlp_uima_repository-1.0.1/docs/api/src-html/edu/uchsc/ccp/util/nlp/document/DocumentSection.html]

```
001    package edu.uchsc.ccp.util.nlp.document;
002    
003    public class DocumentSection {
004    
005            private int documentSectionID;
006            private int sectionStartIndex;
007            private int sectionEndIndex;
008            
009            public int getDocumentSectionID() {
010                    return documentSectionID;
011            }
012            public void setDocumentSectionID(int documentSectionID) {
013                    this.documentSectionID = documentSectionID;
014            }
015            public int getSectionEndIndex() {
016                    return sectionEndIndex;
017            }
018            public void setSectionEndIndex(int sectionEndIndex) {
019                    this.sectionEndIndex = sectionEndIndex;
020            }
021            public int getSectionStartIndex() {
022                    return sectionStartIndex;
023            }
024            public void setSectionStartIndex(int sectionStartIndex) {
025                    this.sectionStartIndex = sectionStartIndex;
026            }
027            
028    }
```
